# Resistance of aerobic microorganisms and soil enzyme response to soil contamination with Ekodiesel Ultra fuel

**DOI:** 10.1007/s11356-017-0076-1

**Published:** 2017-09-10

**Authors:** Agata Borowik, Jadwiga Wyszkowska, Mirosław Wyszkowski

**Affiliations:** 1 Department of Microbiology, University of Warmia and Mazury in Olsztyn, Plac Łódzki 3, 10-727 Olsztyn, Poland; 2 Department of Environmental Chemistry, University of Warmia and Mazury in Olsztyn, Plac Łódzki 4, 10-727 Olsztyn, Poland

**Keywords:** Microbiota, Enzymatic activity, PAHs, Oil products, Degradation, Soil stability

## Abstract

This study determined the susceptibility of cultured soil microorganisms to the effects of Ekodiesel Ultra fuel (DO), to the enzymatic activity of soil and to soil contamination with PAHs. Studies into the effects of any type of oil products on reactions taking place in soil are necessary as particular fuels not only differ in the chemical composition of oil products but also in the composition of various fuel improvers and antimicrobial fuel additives. The subjects of the study included loamy sand and sandy loam which, in their natural state, have been classified into the soil subtype 3.1.1 Endocalcaric Cambisols. The soil was contaminated with the DO in amounts of 0, 5 and 10 cm^3^ kg^−1^. Differences were noted in the resistance of particular groups or genera of microorganisms to DO contamination in loamy sand (LS) and sandy loam (SL). In loamy sand and sandy loam, the most resistant microorganisms were oligotrophic spore-forming bacteria. The resistance of microorganisms to DO contamination was greater in LS than in SL. It decreased with the duration of exposure of microorganisms to the effects of DO. The factor of impact (IF_DO_) on the activity of particular enzymes varied. For dehydrogenases, urease, arylsulphatase and β-glucosidase, it had negative values, while for catalase, it had positive values and was close to 0 for acid phosphatase and alkaline phosphatase. However, in both soils, the noted index of biochemical activity of soil (BA) decreased with the increase in DO contamination. In addition, a positive correlation occurred between the degree of soil contamination and its PAH content.

## Introduction

Progressive industrialisation and urbanisation contribute to the degradation of ever increasing areas, which leads to a reduction in biological diversity (Global Environment Outlook 2012). A significant role in the degradation of natural environments is performed by persistent organic pollutants, including polycyclic aromatic hydrocarbons (Angello et al. 2016). The European Commission, the European Economic and Social Committee and the Committee of the Regions (COM 2006) pay close attention to polycyclic aromatic hydrocarbons (PAHs) as compounds resulting in soil degradation.

The hazards to the soil environment and to human health are not only posed by exhaust emissions from diesel engines, including inter alia PAHs, but also by point contamination of soils with oil products due to failures, accidents, spills during reloading, etc. (Park and Park 2011; Ziółkowska and Wyszkowski 2010). The response of microorganisms and soil enzymes to the effects of particular oil products should be determined individually for particular types and brands of these products (Adam et al. 2017; Niepceron et al. 2013). Not only do they differ in the natural chemical composition but also in the content of improvers, which offer anti-corrosive, cidal, demulsifying, and anti-foam properties and may modify the effects of particular oil products on the quality of soil (Comber et al. 2016; Ramkumar and Kirubakaran 2016). Therefore, a proper assessment of risks to soils contaminated with various hydrocarbons is necessary in order to understand and manage such ecosystems (Pinedo et al. 2012). Furthermore, identifying the metabolic response of soils to particular types of contaminants may facilitate their biotechnological remediation.

Many soil microorganisms are actively involved in restoring the biological balance of soils contaminated with oil products (Table 1). As for bacteria, the following genera are dominant: *Bacillus* (Bento et al. 2005; Fatima et al. 2015), *Pseudomonas* (Fatima et al. 2015), *Staphylococcus* (Moscoso et al. 2012; Silva et al. 2015), *Acinetobacter* (Chang et al. 2011; Mnif et al. 2015) and *Paenibacillus* (Lipińska et al. 2015), while as regards fungi, the following genera predominate: *Aspergillus* (Diaz-Ramirez et al. 2013; El-Hanafy et al. 2017) and *Candida* (Fan et al. 2014; Silva et al. 2015). These products can also be removed from soil by, inter alia, biostimulation of autochthonous microorganisms (Adam et al. 2017; Fan et al. 2014), bioaugmentation (Chang et al. 2011; Fan et al. 2014; Galiulin and Galiulina 2015; Semrany et al. 2012), phytoremediation (Agnello et al. 2016; Sivitskaya and Wyszkowski 2013; Soleimani et al. 2010) and electro-bioremediation (Shrestha et al. 2010; Yuan et al. 2016; Wang et al. 2012). It is important, however, to observe the response of both cultured microorganisms (Kucharski and Jastrzębska 2005; Soleimani et al. 2010; Wyszkowska et al. 2015) to soil contamination and the enzymes of importance in terms of soil quality, i.e. those involved in respiration processes and transformations of carbon, nitrogen, phosphorus and sulphur (Baran et al. 2004; Lipińska et al. 2015; Wu et al. 2014; Wyszkowska et al. 2006). These are the agents which, in the early period following the contamination, indicate the degree of soil degradation (Perez-Leblic et al. 2010; Wu et al. 2014) and facilitate selecting the method of remediation. A good indicator of the biological condition is the measurement of the activity of dehydrogenases (Kaczyńska et al. 2015; Lipińska et al. 2014a). The activity of these enzymes most accurately reflects the reactions taking place in soil as these are intracellular enzymes which means that they are closely related to the microbial activity of soil (Moeskops et al. 2010; Subhani et al. 2001). Urease activity is a rather good indicator as well. Admittedly, it is an extracellular enzyme less related to the condition of microorganisms, yet it is very sensitive to the effects of various xenobiotics (Lipińska et al. 2013; Zhan et al. 2010). An enzyme that is important in the assessment of the reactions taking place in soil is β-glucosidase, which is responsible for the final transformation of cellulose to glucose (Knight and Dick 2004), all the more so as transformations of organic carbon and nitrogen are relatively consistent with each other. The information on disturbances to transformations of organic phosphorus compounds is relatively accurately disclosed by the activity of acid phosphatase and alkaline phosphatase (Kucharski and Jastrzębska 2006; Wyszkowska et al. 2015), and the transformations of organic sulphur are reflected by the activity of arylsulphatase (Lipińska et al. 2014b; Vong et al. 2008). Particular attention should be paid to the relationship between the activity of alkaline phosphatase and the activity of acid phosphatase (Wyszkowska and Wyszkowski 2010). However, in order to avoid randomness in estimating the enzymatic activity of soils, not only does the study assess the activity of particular enzymes but also use the index of activity of soil (BA), which reflects the total activity of all enzymes (Wyszkowska et al. 2013).

**Table 1:** Microorganisms involved in the transformation of oil-derived hydrocarbons

Microorganisms	Reference
Bacteria	
*Pseudomonas*, *Rhodococcus*, *Arthrobacter*	Akbari and Ghoshal (2015)
*Bacillus cereus*, *Bacillus sphaericus*, *B. fusiformis*, *Acinetobacter junii*	Bento et al. (2005)
*Acinetobacter baumannii*	Chang et al. (2011)
*Bacillus cereus*, *Gordoni rubripertincta*, *Kociria rosea*, *Bacillus subtilis*	Diaz-Ramirez et al. (2013)
*Bacillus amyloquefaciens*, *Acinetobacter lwofii*, *Acinetobacter* sp., *Bacillus amyloquefaciens*, *Bacillus cereus*, *Bacillus endophyticus*, *Bacillus flexus*, *Bacillus firmus*, *Bacillus licheniformis*, *Bacillus megaterium*, *Bacillus niabensis*, *Bacillus pumilus*, *Bacillus subtilis*, *Enterobacter cloacae*, *Klebsiella* sp., *Oceanimonas denitrificans*, *Pseudomonas aeruginosa*, *Pseudomonas brassicacearum*, *Shinella granuli*, *Staphylococus sciuri*, *Staphylococus vitulinus*	Fatima et al. (2015)
*Azospirillum* sp., *Pseudomonas stutzeri*	Gałązka et al. (2012)
*Xanthomonas*, *Cupriavidus*, *Phenylobacterium*, *Brevundimonas*, *Caulobacter*	Jung et al. (2016)
*Bacillus frigoritolerans*, *Bacillus simplex*, *Bacillus thuringiensis*, *Bacillus muralis*, *Bacillus pumilus*, *Bacillus safensis*, *Bacillus psychrodurans*, *Bacillus pumilus*, *Bacillus safensis*, *Bacillus aerophilus*, *Bacillus altitudinis*, *Corynebacterium amycolatum*, *Paenibacillus alvei*, *Paenibacillus apiarius*, *Paenibacillus taiwanensis*	Lipińska et al. (2015)
*Bacillus thuringiensis*, *Bacillus weihenstephanensis*, *Acinetobacter radioresistens*	Mnif et al. (2015)
*Staphylococcus warneri*, *Bacillus pumilus*	Moscoso et al. (2012)
*Pseudomonas aeruginosa*, *Escherichia fergusonii*	Pasumarthi et al. (2013)
*Arthrobacter* sp., *Microbacterium arborescens*, *Rhodocyclales bacterium*, *Mycobacterium brumae*, *Chloroflexi bacterium*, *Nocardioides* sp., *Chromatiales bacterium*	Ros et al. (2010)
*Staphylococcus saprophyticus*, *Serratia marcescens*	Silva et al. (2015)
*Pseudomonas veronii*, *Pseudomonas gessardii*, *Comamonas testosteroni*	Wald et al. (2015)
*Bacillus subtilis*	Yengejeh et al. (2014)
*Pseudomonas*, *Rhodococcus*, *Caulobacter*	Yergeau et al. (2012)
*Alcaligenes* sp., *Pseudomonas* sp., *Pandorea* sp., *Paenibacillus*	Thavamani et al. (2012)
*Streptomyces rochei*	Chaudhary et al. (2011)
*Streptomyces intermedius*	Wyszkowska et al. (2002a, b)
Cyanobacteria: *Anabaena fertilissima*	Patel et al. (2016)
Cyanobacteria: *Oscillatoria*, *Phormidium*, *Leptolyngbya*, *Lyngbya*, *Microcoleus*, *Plectonema*, *Nostocs*, *Anabaena*, *Chroococcus*	Soltani et al. (2012)
Fungi	
Mold: *Alternaria alternata*, *Aspergillus terreus*, *Cladosporium sphaerospermum*, *Eupenicillium hirayamae*, *Paecilomyces variotii*	Ameen et al. (2016)
Mold: *Aspergillus terreus*, *Aspergillus carneus*	Díaz-Ramírez et al. (2013)
Mold: *Aspergillus niger*, *Aspergillus oryzae*, *Penicillium commune*	El-Hanafy et al. (2017)
Mold: *Scopulariopsis brevicaulis*, *Graphium tectonae*	Ros et al. (2010)
Yeast: *Candida tropicalis*	Chandran and Das (2011), Fan et al. (2014)
Yeast: *Candida tropicalis*, *Trichosporon asahii*	Gargouri et al. (2015)
Yeast: *Rhodotorula aurantiaca*, *Candida ernobii*	Silva et al. (2015)
Yeast: *Candida digboiensis*	Sood and Lal (2009)

For the above reasons, it was decided to carry out a study aimed at the assessment of the response of soil microorganisms and enzymes to the contamination of soil with Ekodiesel Ultra fuel. A study of this type, conducted on this particular oil product, is innovative.

## Material and methods

### Characteristics of Ekodiesel Ultra fuel

The study tested Ekodiesel Ultra fuel of grade B, which in temperate climates is used from 16 April to 30 September. It is characterised by the following properties: density, 820–845 g dm^−3^; cetane number, min 51; cetane index, min 46; and content: PAHs—max 7% (m/m), solid impurities—max. 24 mg kg^−1^, fatty acid methyl esters—max 7% (*v*/*v*), sulphur—max 10 mg kg^−1^, manganese—max 2 mg dm^−3^ and water—max 200 mg kg^−1^ (www.orlen.pl).

### Soil

**Fig. 1:**
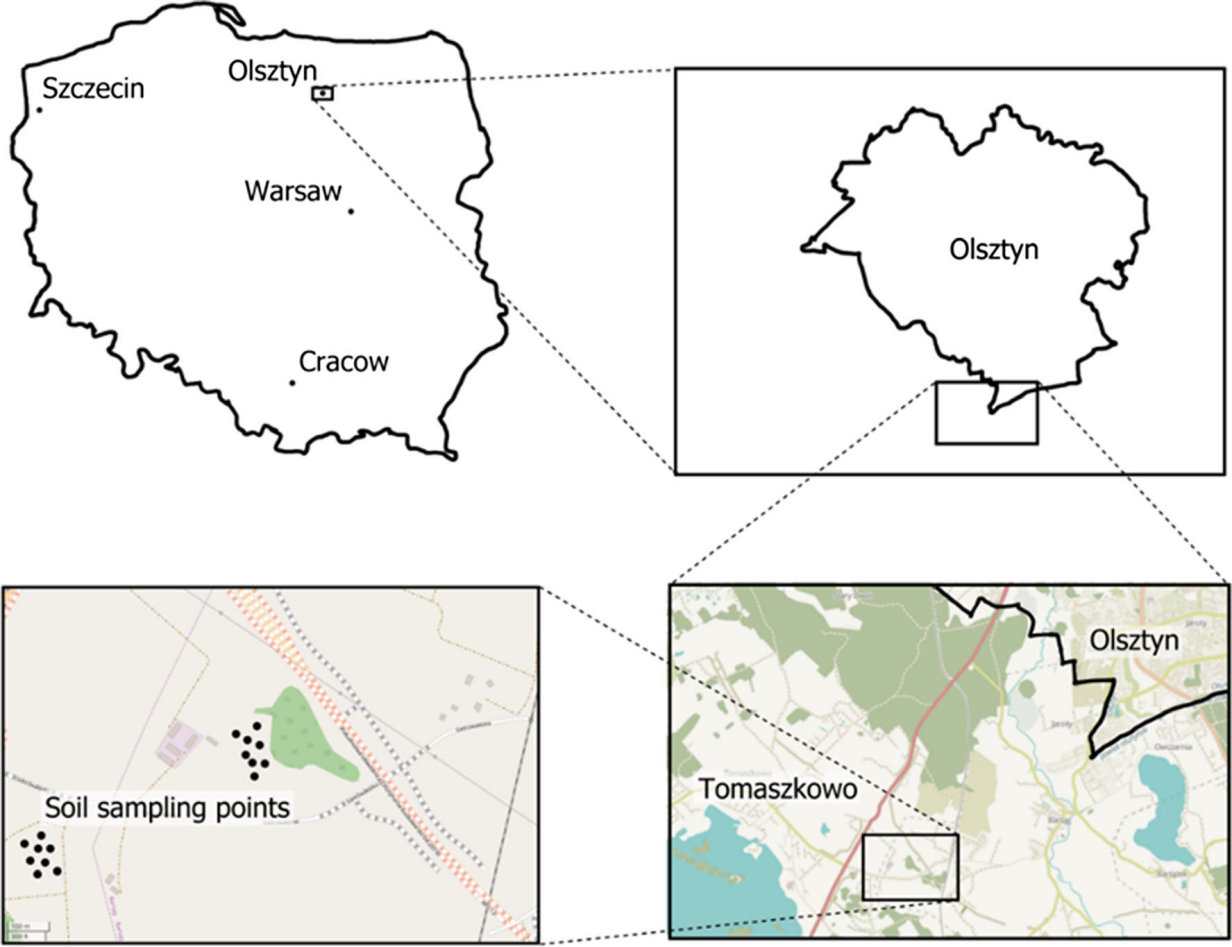
Soil sampling map (https://www.openstreetmap.org 16.08.2017)

The contamination of soils with oil products reduces water capacity and hampers air exchange through filling soil pores and disturbing the C:N ratio. As the destabilisation of physical, chemical and biological properties of soil by diesel fuel is determined by the granulometric composition, the study used two soils significantly differing in the contents of clay, silt and sand (Table 2). For the study, soils typical of the early post-glacial landscape of north-eastern Poland were selected (Fig. 1). To this end, soils used for agricultural purposes, present at the Educational and Experimental Station in Tomaszkowo of the University of Warmia and Mazury in Olsztyn (NE Poland, 53.7167° N, 20.4167° E), were identified. The Educational and Experimental Station in Tomaszkowo is located in the Olsztyn Lakeland (*Pojezierze Olsztyńskie*), a physical and geographical meso-region which is part of the Mazurian Lake District (*Pojezierze Mazurskie*). The soils that predominate there belong to Order 3: Brown earths of the soil type 3.1: Eutric Cambisols. Taking into account the classification in terms of grain size according to the World Reference Base of Soil Resources (2014), for further model study, a soil was selected of the subtype 3.1.1 Endocalcaric Cambisols. The soils were sampled from the tilled Ap horizon (a depth of 0–20 cm). In terms of grain size, they were loamy sand and sandy loam (Table 2).

**Table 2:** Soil characteristics (granulometric composition, physicochemical and chemical properties, microorganisms CFU)

Properties	Kind of soil		Methods of determination
	Loamy sand (LS)	Sandy loam (SL)	
Granulometric composition—% of fractions (*d*, mm)			
Sand 2.00 ≥ *d* > 0.05	75.56a ± 4.54	47.92b ± 2.29	Aerometric PN-R-04032 (1998)
Silt 0.05 ≥ *d* > 0.002	22.92b ± 1.15	48.71a ± 1.70	
Clay *d* ≤ 0.002	1.52b ± 0.08	3.37a ± 0.21	
pH_KCl_	6.7a ± 0.2	6.8a ± 0.2	Potentiometric ISO 10390 (2005)
mmol(+) kg^−1^			
HAC—hydrolytic acidity	7.81b ± 0.41	5.22a ± 0.2	Kappen Klute (1996)
EBC—exchangeable base cations	98.72b ± 4.89	131.41a ± 6.01	
CEC—cation exchange capacity	106.53b ± 5.18	136.63a ± 6.08	
%			
BS—base saturation	92.67b ± 3.12	96.18a ± 3.38	
Content—g kg^−1^			
C_organic_	11.01a ± 0.52	9.92b ± 0.41	Tiurin Nelson and Sommers (1996)
N_total_	0.97b ± 0.05	1.14a ± 0.06	Kjeldahl ISO 11261 (1995)
Content mg kg^−1^			
P_available_	44.92b ± 1.41	47.94a ± 1.24	Egner-Riehm Egner et al. (1960)
K_available_	75.21a ± 3.87	69.13b ± 3.38	
Mg_available_	21.93a ± 1.13	12.74b ± 0.51	Atomic absorption spectrometry Schlichting et al. (1995)
Number of microorganisms—CFU 10^*n*^ kg^−1^			
Oligotrophic bacteria	14.53a ± 2.18	6.84b ± 1.03	Substrates and abbreviations of microbial names are presented in Table 3
Oligotrophic sporulating bacteria	5.72a ± 0.80	4.75a ± 0.66	
Copiotrophic bacteria	10.11a ± 1.36	2.68b ± 0.36	
Copiotrophic sporulating bacteria	4.88a ± 0.49	5.53a ± 0.57	
*Azotobacter*	1.04b ± 0.12	5.53a ± 0.64	
*Arthrobacter*	10.24a ± 1.52	9.00a ± 1.33	
*Pseudomonas*	11.70b ± 1.76	26.02a ± 3.90	
Ammonifying bacteria	9.33a ± 1.52	10.96a ± 1.79	
Nitrogen immobilisation bacteria	12.55a ± 1.07	10.24b ± 1.19	
Cellulolytic bacteria	17.88a ± 3.06	3.93b ± 0.67	
Actinomycetes	9.65b ± 1.02	11.52a ± 0.81	
Fungi	4.29b ± 0.64	6.38a ± 0.96	

*n*—exponent: 8 for oligotrophic sporulating and copiotrophic sporulating bacteria; 7 for cellulolytic bacteria and Fungi; 4 for *Azotobacter*; 8 for *Arthrobacter* and *Pseudomonas*; 9 for other microorganisms. The same lowercase letters in the rows indicate homogeneous groups

### Experimental design

Since oil products have great potential for accumulation in the soil environment and may lead to gradual degradation of soils, it is particularly important that the changes to be expected under field conditions should be observed, in the first place, in specially designed model experiments conducted under controlled conditions. With this principle in mind, the authors of the study using Ekodiesel Ultra fuel carried out an experiment under monitored conditions, in a greenhouse, in polyethylene pots with a capacity of 3.5 dm^3^, in 4 repetitions. In the experiment, the variable factors included the following:

- Type of soil formation: loamy sand and sandy loam
- The degree of the contamination of soil with Ekodiesel Ultra fuel in cubic centimeters per kilogram of soil DM: 0, 5 and 10
- The duration of incubation of soil samples, in days: 30 and 60

Prior to the establishment of the experiment, the selected and precisely pre-characterised soils were passed through a 2-mm sieve. The soil samples (each weighing 3 kg) were then mixed with Ekodiesel Ultra fuel and brought to a moisture content corresponding to 60% of the capillary water capacity. The moisture content of soil was monitored and maintained at a constant level throughout the duration of the experiment, i.e. for 60 days. In order to maintain the soil moisture content at a level of 60% of the capillary water capacity, soil in the pots was covered with perforated foil and weighed once a week and any water losses were replenished. A separate series of soils was prepared for each date of testing.

The experiment was conducted in 2015, in the months June–July. The monthly average temperature was 15.5 °C in June and 17.9 °C in July. After 30 and 60 days of the experiment, soil samples were transported to a laboratory and kept at a temperature of 4 °C. Microbiological analyses were performed directly after taking the soil samples, and biochemical determinations were made on the following day. The physicochemical properties of soil were determined in air-dry samples stored in the dark for 10 days.

### Determination of the soil microorganism number

On days 30 and 60 of the experiment, soil samples were collected for analyses, and the next stage of the study was commenced in a laboratory. Microbiological analyses were first conducted using the soil culture dilution method. Soil samples (10 g) were weighed to a sterile physiological saline solution (90 cm^3^ of 0.85% NaCl) and shaken for 30 min at 120 rpm. Two appropriate dilutions of soil culture were inoculated into Petri dishes in parallel in four repetitions: oligotrophic bacteria (Olig), copiotrophic bacteria (Cop), nitrogen-immobilising bacteria (Im), ammonifying bacteria (Am) and actinobacteria (Act)—dil. of 10^−5^ and dil. of 10^−6^, *Arthrobacter* (Art), *Pseudomonas* (Ps)—dil. of 10^−4^ and dil. of 10^−5^, oligotrophic spore-forming bacteria (Olig_p_) and copiotrophic spore-forming bacteria (Cop_p_)—dil. of 10^−3^ and dil. of 10^−4^, cellulolytic bacteria (Cel), and fungi (Fun)—dil. of 10^−3^ and dil. of 10^−4^ and *Azotobacter* spp. (Az)—dil. of 10^0^. Appropriate selective mediums were then introduced. The composition of microbiological mediums on which microorganisms were cultured is presented in Table 3. Microorganisms were cultured on Petri dishes at a temperature of 28 °C, within a period ranging from 2 (*Azotobacter*) to 21 days (oligotrophic bacteria). The spore forms of oligotrophic and copiotrophic bacteria were determined in the material which was pasteurised for 15 min at a temperature of 85 °C. The number of colony forming units (CFU) was determined using a colony counter.

**Table 3:** Medium for determination of number of soil microorganisms

Microorganisms	Medium	References
Olig—oligotrophic bacteria Olig_p_—oligotrophic spore-forming bacteria	Peptone 0.10 g; meat extract 0.10 g; NaCl 0.05 g; agar 10.0 g; H_2_O 1.0 dm^3^; pH 7.0–7.2	Ohta and Hattori (1983)
Cop—copiotrophic bacteria Cop_p_—copiotrophic spore-forming bacteria	Peptone 10 g; meat extract 10 g; NaCl 5 g; agar 10.0 g; H_2_O 1.0 dm^3^; pH 7.0–7.2	Ohta and Hattori (1983)
Az—*Azotobacter*	K_2_HPO_4_ 1.5 g; MgSO_4_⋅7H_2_O 0.3 g; NaCl 0.3 g; FeSO_4_⋅7H_2_O 0.005 g; MnSO_4_⋅7H_2_O 0.005 g; CaCO_3_ 3.0 g; saccharose 15.0 g; agar 7.0 g; H_2_O 1.0 dm^3^; pH 7.0–7.2	Fenglerowa (1965)
Art—*Arthrobacter*	CaH_2_PO_4_ 0.25 g; K_2_HPO_4_ 1.0 g; MgSO_4_⋅7H_2_O 0.25 g; glycerol 10 cm^3^; agar 14.0 g; H_2_O 1.0 dm^3^; pH 7.0	Mulder and Antheumisse (1963)
Ps—*Pseudomonas*	Peptone 20 g; K_2_HPO_4_ 1.5 g; MgSO_4_⋅7H_2_O 1.5 g; agar 14.0 g; glycerol 10 cm^3^; H_2_O 1.0 dm^3^; pH 7.2	Mulder and Antheumisse (1963)
Am—ammonifying bacteria	Peptone 5 g; K_2_HPO_4_ 0.5 g; MgSO_4_ 0.2 g; NaCl 0.2 g; MnSO_4_ 0.005 g; FeSO_4_ 0.005 g; NH_4_)_2_Fe(SO_4_)_2_⋅6H_2_O 0.005 g; agar 14 g; nystatin 7.5 mg·1 dm^−3^ medium; H_2_O 1.0 dm^3^; pH 7.0–7.2	Winogradski (1953)
Im—nitrogen immobilisation bacteria	K_2_HPO_4_ 0.5 g; MgSO_4_ 0.2 g; NaCl 0.2 g; MnSO_4_ 0.005 g; FeSO_4_ 0.005 g; (NH_4_)_2_Fe(SO_4_)_2_⋅6H_2_O 0.005 g; saccharose 0 g; NH_4_NO_3_ 0.2 g; agar 14 g; H_2_O 1.0 dm^3^; pH 7.0–7.2	Winogradski (1953), our modification—instead of (NH_4_)_2_SO_4_ used NH_4_NO_3_
Cel—cellulolytic bacteria	K_2_HPO_4_ 0.5 g; MgSO_4_ 0.2 g; NaCl 0.2 g; MnSO_4_ 0.005 g; FeSO_4_ 0.005 g; KNO_3_ 1.0 g; (NH_4_)_2_Fe(SO_4_)_2_⋅6H_2_O 0.005 g; agar 14 g; H_2_O 1.0 dm^3^; pH 7.0–7.2	Winogradski (1953)
Act—actinomycetes	Soluble starch 10.0 g; casein 0.3 g; KNO_3_ 2.0 g; NaCl 2.0 g; K_2_HPO_4_ 2.0 g; MgSO_4_⋅7H_2_O 0.05 g; CaCO_3_ 0.02 g; FeSO_4_ 0.01 g; agar 20.0 g; H_2_O 1 dm^3^; 50 cm^3^ aqueous solution of nystatin 0.05%; 50 cm^3^ aqueous solution of actidione 0.05%; pH 7.0	Parkinson et al. (1971)
Fun—fungi	Peptone 5 g; K_2_HPO_4_ 1.0 g; glucose 10 g; MgSO_4_⋅7H_2_O 0.5 g; agar 20.0 g; H_2_O 1 dm^3^; 3.3 cm^3^ aqueous solution of bengal rose 1%; 25 cm^3^ aqueous solution of aureomycin 0.01%; pH 5.9	Martin (1950)

### Determination of the activity of soil enzymes

At the same time that the number of microorganisms was determined, i.e. on days 30 and 60 of the experiment in soil samples, from each repetition in three subsequent replications, the activity of dehydrogenases, catalase, urease, acid phosphatase, alkaline phosphatase, β-glucosidase and arylsulphatase was determined. The substrates used for the determination of the enzyme activity, as well as the units in which the activity of particular enzymes was expressed, are presented in Table 4. The activity of all enzymes, with the exception of catalase, was determined using a Perkin-Elmer Lambda 25 spectrophotometer (MA, USA). The activity of dehydrogenases was determined at a wavelength (*λ*) of 485 nm; the activity of urease, acid phosphatase and alkaline phosphatase at 410 nm; the activity of β-glucosidase at 400 nm; and the activity of arylsulphatase at 420 nm. The activity of catalase was determined based on the reaction of hydrogen peroxide decomposition using potassium permanganate.

**Table 4:** Methods of determination of soil enzyme activity

Enzyme	Substrate	Product/unit	References
Deh—dehydrogenases (EC 1.1)	2,3,5-Triphenyl tetrazolium chloride (TTC)	Triphenyl fomazan (TFF), μmol kg^−1^ DM of soil h^−1^	Öhlinger (1996)
Cat—catalase (EC 1.11.1.6)	H_2_O_2_—aqueous solution	O_2_, mol kg^−1^ DM of soil h^−1^	Alef and Nannipieri (1998)
Ure—urease (EC 3.5.1.5)	Urea—aqueous solution	N-NH_4_, mmol kg^−1^ DM of soil h^−1^	Alef and Nannipieri (1998)
Glu—β-glucosidase (EC 3.2.1.21)	4-Nitrophenyl-β-D-glucopyranoside (PNG)	4-Nitrophenol (PN), mmol kg^−1^ DM of soil h^−1^	Alef and Nannipieri (1998)
Pac—acid phosphatase (EC 3.1.3.2)	Disodium 4-nitrophenyl phosphate hexahydrate (PNP)	4-Nitrophenol (PN), mmol kg^−1^ DM of soil h^−1^	Alef and Nannipieri (1998)
Pal—alkaline phosphatase (EC 3.1.3.1)	Disodium 4-nitrophenyl phosphate hexahydrate (PNP)	4-Nitrophenol (PN), mmol kg^−1^ DM of soil h^−1^	Alef and Nannipieri (1998)
Aryl—aryosulphatase (EC 3.1.6.1)	Potassium-4-nitrophenylsulfate (PNS)	4-Nitrophenol (PN), mmol kg^−1^ DM of soil h^−1^	Alef and Nannipieri (1998)

### Determination of PAH content

In soil samples (loamy sand and sandy loam) contaminated with Ekodiesel Ultra fuel, after 60 days, the contents of 9 PAHs were determined, i.e. naphthalene (NAP), phenanthrene (PHE), anthracene (ANT), fluoranthene (FTH), benzo(*a*)antracene (BaA), chrysene (CHR), benzo(*a*)fluoranthene (BaF), benzo(*a*)pyrene (BaP) and benzo(*ghi*)perylene (BghiP). The reference was the determination of PAH contents in samples of non-contaminated soil. The content of polycyclic aromatic hydrocarbons was determined using an Agilent 7890A gas chromatograph coupled with Agilent 5975C mass spectrometer equipped with an EI/CI ion source. The hydrocarbons were extracted in accordance with the standard ISO 18287 (2006).

### Determination of physicochemical and chemical properties of the soil

Prior to the establishment of the experiment, and on day 60 of the experiment, soil samples were taken (from each pot) and then dried and passed through a 2-mm sieve. In the prepared soil, the following were then determined: granulometric composition of soil, pH value of soil, hydrolytic acidity (HAC) and exchangeable base cations (EBC). Based on the HAC and EBC values, the sorption capacity of soil (CEC) and the degree of soil saturation with basic elements (BS) were determined. The following formulas were applied: CEC = EBC + HAC; BS = (EBC/CEC)·100. The contents of total nitrogen, organic carbon (C_org_), available phosphorus, potassium and magnesium were also determined. The methods applied to determine the physicochemical and chemical properties are presented in Table 2.

### Methodology of calculations

The varied effects of Ekodiesel Ultra fuel on the soil microbiota are presented using the index of resistance of microorganisms (RS), the factor of impact of diesel fuel (IF_DO_) and the index of biochemical activity of soil (BA). Taking into account the number of 12 groups of microorganisms, their resistance to the effects of diesel fuel was calculated. To this end, the formula provided by Orwin and Wardle (2004) was applied:

1 $$ \mathrm{RS}=1-\frac{2\ \left|\ {D}_0\left.\right|\right.}{C_0+\left|\ {D}_0\left.\right|\right.} $$

RS: resistance.
*C*_0_: a value of the tested parameter for control soil.
*P*_0_: a value of the tested parameter for contaminated soil.
*D*_0_ : *C*_0_ − *P*_0_.

The RS index takes values from − 1 to + 1. RS with a value of 1 means full resistance; 0—a 100% decrease, or a 100% increase of the tested characteristic; negative values—an increase by more than 100%, the more the RS is close to − 1, the greater the increase—by more than 100%.

The factor of impact of diesel fuel (IF_DO_) on the activity of soil enzymes was calculated according to formula no. 2 and the index of biochemical activity of soil (BA) was calculated based on formula no. 3 (Wyszkowska et al. 2013).

2 $$ {\mathrm{IF}}_{\mathrm{DO}}=\frac{{}_{P_0-{C}_0}}{C_0} $$

IF_DO_—DO impact factor.

*C*_0_ and *P*_0_—designations are provided in formula No 1.

If IF_DO_ = 0—no impact, − 1—100% inhibition, + 1—100% stimulation.

3 $$ \mathrm{BA}=\mathrm{Deh}+\mathrm{Kat}+\mathrm{Pal}+\mathrm{Pac}+\mathrm{Ure}+\mathrm{Glu}+\mathrm{Aryl} $$

BA: biochemical activity.
Deh: the activity of dehydrogenases (μmol TFF kg^−1^ DM h^−1^).
Kat: the activity of catalase (mol O_2_ kg^−1^ DM h^−1^).
Ure: the activity of urease (mmol N-NH_4_^+^ kg^−1^ DM h^−1^).
Glu: the activity of β-glucosidase (mmol PNP kg^−1^ DM h^−1^).
Pac: the activity of acid phosphatase (mmol PNP kg^−1^ DM h^−1^).
Pal: the activity of alkaline phosphatase (mmol PNP kg^−1^ DM h^−1^).
Aryl: the activity of arylsulphatase (mmol PNP kg^−1^ DM h^−1^).

### Statistical analysis

The study results were analysed statistically. In the interpretation of the effects of Ekodiesel Ultra fuel on shaping the number of microorganisms and the enzymatic activity, it was helpful to determine the percentages of particular independent variables in shaping the dependent variables. To this end, an analysis of the measure of the effect *η* ^2^ was applied and carried out using the variance analysis ANOVA method. The homogeneous groups were counted using Tukey’s test at *P* = 0.01. In the assessment of the effects of Ekodiesel Ultra fuel on microorganisms and soil enzymes, cluster analysis (CA) was applied. In order to estimate the distance between clusters, variance analysis was applied. The distance between clusters was measured using Euclidean distance by Ward’s method. The results were also analysed using a principal component analysis (PCA) test, and correlation coefficients were calculated between the PCA results. In the statistical processing of results, a Statistica 12.5 package (StatSoft, Inc. 2015) was used.

## Results

### Soil microorganisms

**Fig. 2:**
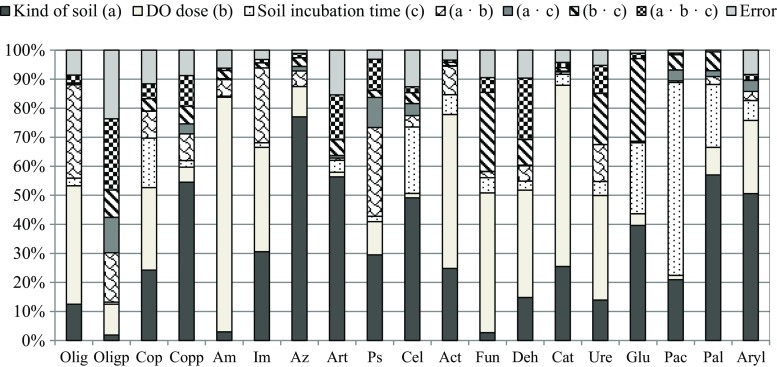
Percent of the observed variability *η* ^2^ in shaping the number of soil microorganisms and activity of soil enzymes. Explanations are provided in Tables 3 and 4. DO—Ekodiesel Ultra fuel

Initial CFU of microorganisms in soil were included in Table 2. Of the three tested factors, i.e. the type of soil, the degree of contamination with Ekodiesel Ultra fuel and the duration of incubation, the number of soil microorganisms was most significantly affected by the first two factors (Fig. 2). Their participation in shaping the number certainly varied for particular groups and genera of microorganisms. The type of soil determined, to the greatest extent, the number of the following bacteria: *Azotobacter* sp. (77%), *Arthrobacter* sp. (56%), copiotrophic spore-forming bacteria (55%), cellulolytic bacteria (49%), nitrogen-immobilising bacteria (31%), *Pseudomonas* sp. (29%), actinobacteria (25%) and total copiotrophic bacteria (24%). The type of soil affected other groups of microorganisms to a much lesser degree. The impact of this factor ranged from 2% (Olig_sp_) to 12% (Olig). In turn, the contamination of soil with Ekodiesel Ultra fuel had the greatest effect on the number of the following: ammonifying bacteria (81%), actinobacteria (53%), oligotrophic bacteria (41%), nitrogen-immobilising bacteria (36%), copiotrophic bacteria (28%) and fungi (48%). The number of other groups of microorganisms was to a much lesser degree determined by the contamination with Ekodiesel Ultra fuel, and the percentage of this contaminant in the structure of the number ranged from 2% (cellulolytic bacteria) to 11% (*Pseudomonas* sp.).

**Fig. 3:**
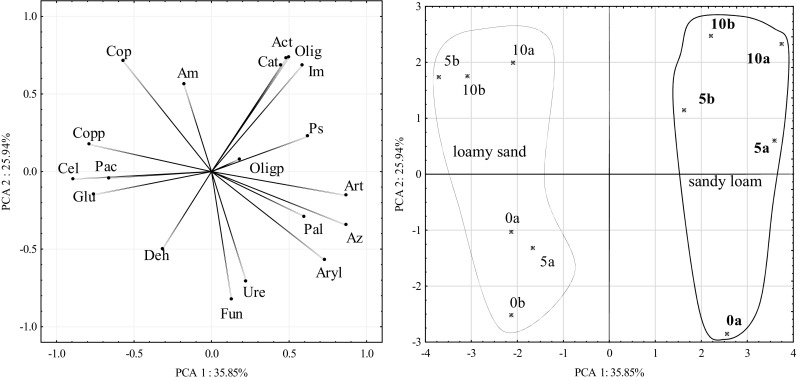
Number of microorganisms and enzymatic activity of soil contaminated with Ekodiesel Ultra fuel (DO) represented with the PCA. Kind of soil: SL—sandy loam; LS—loamy sand; DO dose (cm^3^ kg^−1^ DM of soil): 0, 5, 10; analysis term: a—30 days, b—60 days. Explanations are provided in Tables 3 and 4

**Fig. 4:**
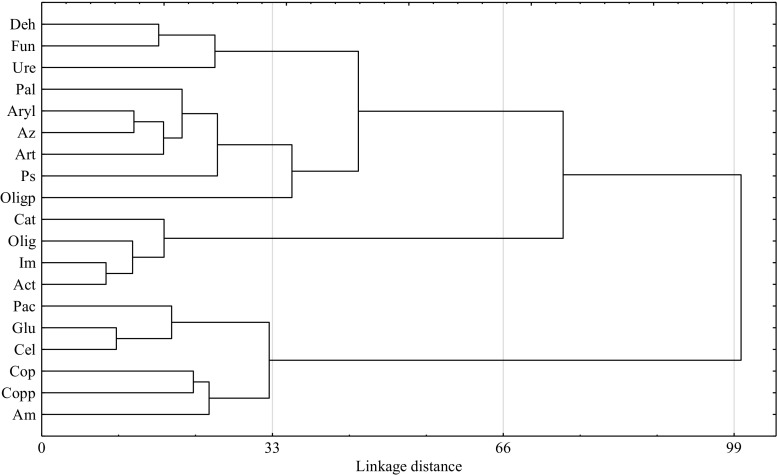
Similar response of microorganisms and enzyme activity in soil contaminated with Ekodiesel Ultra fuel (DO). Explanations are provided in Tables 3 and 4

Therefore, the response of microorganisms to the contamination of soil with Ekodiesel Ultra fuel varied (Fig. 3). A significant correlation occurred between the number of *Arthrobacter* sp. and *Azotobacter* sp. (*r* = 0.52, *P* = 0.001), actinobacteria and oligotrophic bacteria (*r* = 0.64, *P* < 0.001), actinobacteria and nitrogen-immobilising bacteria (*r* = 0.88, *P* < 0.001), oligotrophic and nitrogen-immobilising bacteria (*r* = 0.70, *P* < 0.001), total copiotrophic bacteria and ammonifiers bacteria (*r* = 0.37, *P* = 0.026) and cellulolytic and copiotrophic spore-forming bacteria (*r* = 0.62, *P* < 0.001). Such a relationship is not only proven by the data presented using the PCA method but also by the similarity of responses of particular groups of microorganisms, documented by Ward’s cluster method (Fig. 4).

The greatest increase in the number (Fig. 3) of actinobacteria, as well as oligotrophic and nitrogen-immobilising bacteria, was observed in sandy loam contaminated with 10 cm^3^ of DO, irrespective of the duration of soil incubation, while the greatest increase in the number of copiotrophic bacteria and their spore forms, ammonifying and cellulolytic bacteria, was observed in loamy sand contaminated with 10 cm^3^ of DO on both dates of the study and in loamy sand contaminated with 5 cm^3^ of DO on the second date of the study. In the mentioned objects, a negative response of bacteria of the *Arthrobacter* and *Azotobacter* genera to the contamination with DO occurred. The response of fungi to contamination with DO was unequivocally negative in both soils, irrespective of the date of testing.

**Fig. 5:**
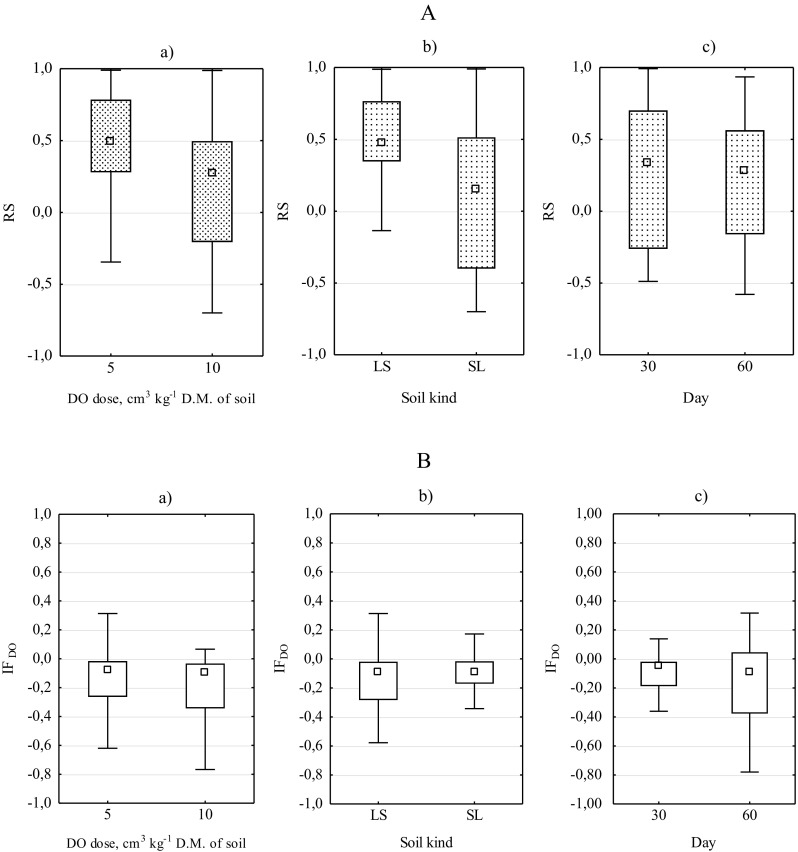
Resistance (**a**) of microorganisms (RS) and impact factor (**b**) of diesel oil (IF_DO_) on biochemical activity of soil (BA) to soil contaminated with Ekodiesel Ultra fuel (DO) depending on (a) diesel oil dose, (b) kind of soil and (c) soil incubation time

**Fig. 6:**
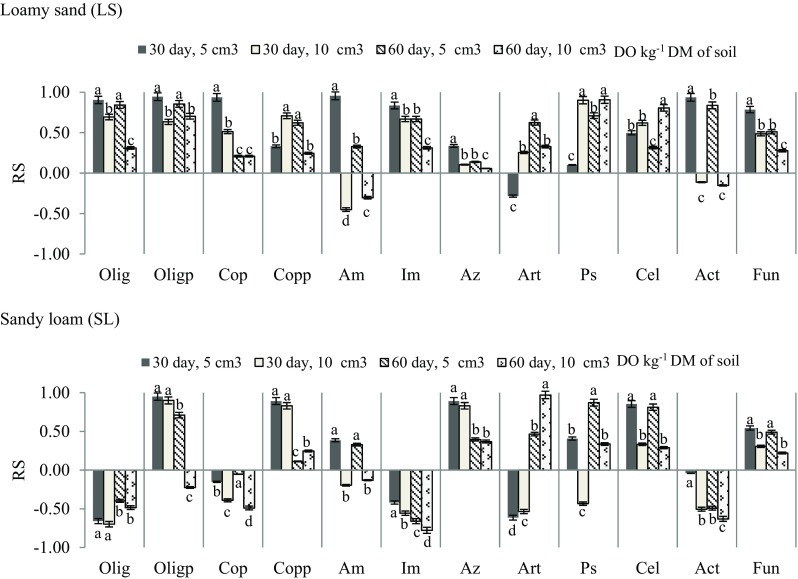
Resistance of microorganisms (RS) to soil contaminated with Ekodiesel Ultra fuel (DO) depending on the kind of soil. Explanations are provided in Tables 3 and 4. Identical lowercase letters for soil microorganisms are assigned to homogeneous groups (*P* < 0.01)

In general, the lower the average resistance of microorganisms to the effects of DO was, the greater the contamination with this pollutant was, i.e. it was lower in sandy loam than in loamy sand; moreover, it was related to the smallest extent to the date of testing (Fig. 5). There is certainly a significant difference between the resistances of particular groups. In loamy sand, the most resistant bacteria (Fig. 6) were oligotrophic spore-forming bacteria (RS = 0.63–0.95), while the least resistant were bacteria of the *Azotobacter* genus (RS = 0.06–0.34). In sandy loam, the most resistant bacteria were oligotrophic spore-forming bacteria (RS = 0.71–0.95), while the least resistant were bacteria of the nitrogen-immobilising bacteria (RS = − 0.42 to − 0.78). With time, the resistance of both spore-forming groups decreased.

### Physicochemical properties

The impact of fuel contamination on physicochemical properties was minimal (Table 5). In both loamy sand and sandy loam, the amount of organic carbon in contaminated objects increased. The pool of total nitrogen and available potassium did not change, while the contents of available phosphorus and magnesium only changed in loamy sand. The soil pH and hydrolytic acidity were stable. In loamy sand, under the effect of Ekodiesel Ultra fuel, the total content of exchangeable cations and the exchange capacity increased, while the degree of soil saturation with alkaline cations of both soils remained unchanged.

**Table 5:** The effect of diesel oil on the physicochemical properties of the soil

DO dose cm^3^ kg^−1^	C_org_	N_og_	P	K	Mg	C:N	C:P	N:P	pH_KCl_	HAC	EBC	CEC	BS [%]
	g kg^−1^ DM of soil		mg kg DM of soil							mmol(+) kg^−1^ DM of soil			
Loamy sand (LS)													
0	11.1b ± 0.5	1.0b ± 0.05	45.5b ± 1.4	75.8a ± 3.9	22.0a ± 1.1	11.1	244.0	22.0	6.7a ± 0.1	8.4a ± 0.3	106.0c ± 4.9	114.4c ± 3.7	92.7b ± 4.3
5	11.9a ± 0.6	1.0b ± 0.04	43.6c ± 1.8	75.9a ± 3.7	17.6b ± 1.2	11.9	272.9	22.9	6.7a ± 0.2	8.6a ± 0.4	115.7b ± 4.5	124.3b ± 4.6	93.1b ± 3.7
10	12.1a ± 0.6	1.0b ± 0.04	43.1c ± 1.8	79.5a ± 4.1	16.4b ± 1.3	12.1	280.7	23.2	6.7a ± 0.2	8.3a ± 0.3	116.7b ± 5.2	125.0b ± 5.4	93.4b ± 3.2
Sandy loam (SL)													
0	10.3c ± 0.4	1.1a ± 0.05	47.9a ± 1.2	69.1b ± 3.4	12.7c ± 0.5	9.4	215.0	23.0	6.8a ± 0.2	5.3b ± 0.2	137.7a ± 5.3	143.0a ± 6.4	96.3a ± 4.2
5	10.8b ± 0.4	1.2a ± 0.04	48.0a ± 1.4	68.2b ± 3.1	12.3c ± 0.8	9.0	225.0	25.0	6.8a ± 0.2	5.5b ± 0.4	138.1a ± 4.6	143.6a ± 6.9	96.2a ± 4.0
10	11.1b ± 0.4	1.2a ± 0.05	48.1a ± 1.7	70.8b ± 2.9	12.6c ± 0.4	9.3	230.8	24.9	6.8a ± 0.2	5.1b ± 0.4	138.6a ± 6.1	143.7a ± 7.1	96.4a ± 3.4

Identical lowercase letters in columns are assigned to homogeneous groups (*P* < 0.01). Explanations are provided in Table 2

DO—Ekodiesel Ultra fuel

### Soil enzymes

The impact degree of the tested independent variables of the activity of soil enzymes varied, similar to the impact degree on the activity of soil microorganisms (Fig. 2). The Ekodiesel Ultra fuel had the strongest effect on the activity of dehydrogenases, catalase, urease and arylsulphatase and the weakest effect on the activity of acid phosphatase, β-glucosidase and alkaline phosphatase. The activity of dehydrogenases was determined in 37% by the contamination of soil with Ekodiesel Ultra fuel (catalase in 62%; urease in 36%; arylsulphatase in 25%; acid phosphatase in 1.5%; β-glucosidase in 4%; and alkaline phosphatase in 10%). The percentage participation of the type of soil in shaping the activity of enzymes ranged from 14% (urease) to 57% (alkaline phosphatase), whereas the duration of incubation ranged from 3 to 7% (dehydrogenases, catalase, urease, arylsulphatase), 22–24% (alkaline phosphatase, β-glucosidase) and to 66% (acid phosphatase).

A PCA demonstrated that the response of dehydrogenases to Ekodiesel Ultra fuel was inverse to the response of catalase (Fig. 3). Dehydrogenases, acid phosphatase and β-glucosidase were strongly correlated with one another, while the other group comprised urease, arylsulphatase and alkaline phosphatase. This results from both the data shown in Fig. 3 and from the data shown in Fig. 4, which presents the relationship between the activities of enzymes using Ward’s cluster analysis method.

**Fig. 7:**
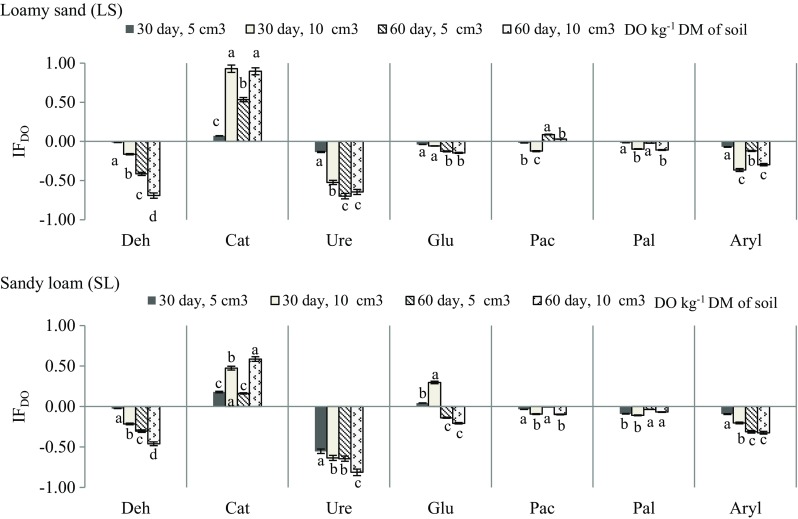
Impact factor (IF_DO_) of Ekodiesel Ultra fuel (DO) on activity of soil enzymes depending on the kind of soil. Explanations are provided in Tables 3 and 4. Identical lowercase letters for soil enzymes are assigned to homogeneous groups (*P* < 0.01)

Catalase was the only enzyme that was stimulated by Ekodiesel Ultra fuel. Such an effect was noted in both soils, on both dates of testing. The impact factor (IF_DO_) had positive values for catalase, both in loamy sand and in sandy loam and ranged from 0.07 to 0.93 in the first soil and from 0.16 to 0.59 in the second soil (Fig. 7). Negative values of IF_DO_ were noted for the remaining enzymes. The greatest inhibition of the activity occurred for urease (IF_DO_ = − 0.14 to − 0.81), dehydrogenases (IF_DO_ = − 0.01 to − 0.62) and arylsulphatase (IF_DO_ = − 0.07 to − 0.37). The activity of other enzymes was inhibited to a much lesser degree. Such tendencies persisted during both parts of the study.

**Fig. 8:**
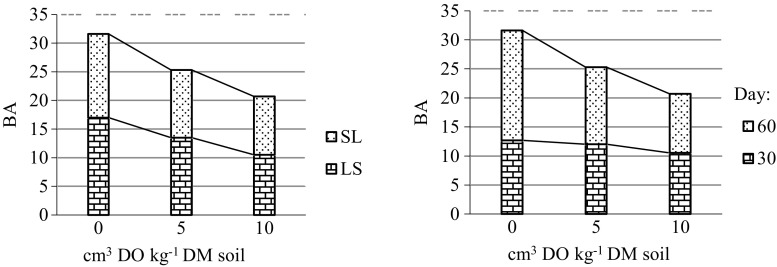
Effect of Ekodiesel Ultra fuel (DO) on biochemical activity of soil (BA)

Irrespective of the stimulating effect of the Ekodiesel Ultra fuel on catalase, the quality of loamy sand and sandy loam, measured using the BA index, significantly decreased (Fig. 8). The impact factor (IF_DO_) on the BA value was negative on both dates of testing, and it became increasingly negative with greater soil contamination (Fig. 5).

### PAHs

**Fig. 9:**
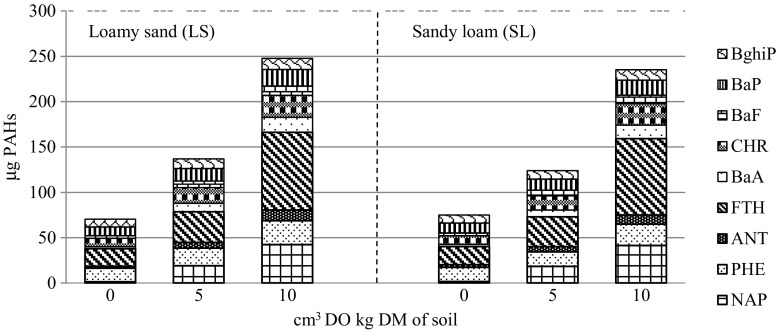
The effect of Ekodiesel Ultra fuel (DO) on the content of polycyclic aromatic hydrocarbons in the soil on day 60 of the experiment, μg PAHs kg^−1^ DM of soil. NAP—naphthalene, PHE—phenanthrene, ANT—anthracene, FTH—fluoranthene, BaA—benzo(*a*)antracene, CHR—chrysene, BaF—benzo(*a*)fluoranthene, BaP—benzo(*a*)pyrene, BghiP—benzo(*ghi*)perylene

The contamination of soil with Ekodiesel Ultra fuel increased the PAH content of both tested soils (Fig. 9). In loamy sand, under the effect of Ekodiesel Ultra fuel at an amount of 5 cm^3^ kg^−1^, a 94% increase in the total PAH content occurred, while under the effect of an amount of 10 cm^3^ kg^−1^, an increase by up to 252% was observed; in sandy loam contaminated with the lower amount of Ekodiesel Ultra fuel, an increase in PAH content by 65% occurred, while for the higher amount, it was 214%. The concentration of naphthalene under the effect of 10 cm^3^ of Ekodiesel Ultra fuel increased in loamy sand by 33 times, and in sandy loam, by 28 times; for benz(*a*)anthracene, by six and five times, respectively; for anthracene, by four and two times; for benzo(*a*)pyrene, by 1.9 and 1.6; for fluoranthene, by four times in both soils; and for benzo(*a*)fluoranthene, by three times, also in both soils. Despite the significant increase in aromatic hydrocarbon content of soils resulting from the contamination with Ekodiesel Ultra fuel, the concentration of each hydrocarbon did not exceed the acceptable standards. The soils contaminated with Ekodiesel Ultra fuel contained the most fluoranthene (86 μg kg^−1^ DM) and the least benzo(*a*)fluoranthene (9 μg kg^−1^ DM).

## Discussion

### Effect of diesel oil and soil physicochemical properties on soil microorganisms

The response of microorganisms to soil contamination with Ekodiesel Ultra fuel is not clear. Not only was it determined by the physiological group of microorganisms and by their genus but also by the type of soil. The type of soil produces effects, not only as a specific living place, but also acts on microorganisms as a protective buffer (Griffiths and Philippot 2013). In our study, Ekodiesel Ultra fuel changes values of EBC and CEC only in loamy sand. Sandy clay, being a more buffered soil, was more resistant to the impact of Ekodiesel Ultra fuel and the analysed soil properties maintained similar values, regardless of the contamination. Highly positive correlation with the soil sorption capacity was demonstrated for the bacteria *Azotobacter* (*r* = 0.79, *P* = 0.002) and *Arthrobacter* (*r* = 0.61, *P* = 0.035), whereas cellulolytic bacteria were highly negatively correlated with this characteristic (*r* = − 0.72, *P* = 0.009). Negative correlations were determined between the HAC value and the following bacteria: *Azotobacter* (*r =* − 0.86, *P* < 0.001), nitrogen-fixing (*r* = − 0.59, *P* = 0.045) and *Arthrobacter* (*r* = − 0.58, *P* = 0.045). In soil polluted with Ekodiesel Ultra fuel, significant positive correlations between the C:N ratio and groups of microorganisms were determined only for sporulating copiotrophic bacteria (*r* = 0.61, *P* = 0.035) and ammonifying bacteria (*r* = 0.62, *P* = 0.032), while significant negative correlations were detected between the C:N ratio and *Azotobacter* (*r* = − 0.62, *P* = 0.032). There were positive interactions between the C:P ratio and copiotrophic bateria (*r =* 0.58, *P* = 0.050), copiotrophic sporulating bacteria (*r =* 0.60, *P* = 0.041) and amonifying bacteria (*r =* 0.69, *P* = 0.014). Also, a positive correlation was observed between the N:P ratio and oligotrophic bacteria (*r* = 0.79, *P* = 0.002), nitrogen-fixing bacteria (*r* = 0.80, *P* = 0.002), *Arthrobacter* (*r* = 0.74, *P* = 0.006), *Pseudomonas* (*r* = 0.60, *P* = 0.039) and *Actinomyces* (*r* = 0.79, *P* = 0.002). Noteworthy is a very weak correlation between the analysed microorganisms and soil sorption capacity, which is a factor which contributes the most to the soil’s availability of nutrients to microorganisms (Canbolat et al. 2006). The most probable reason was the interference of the contamination with the physical properties of the soil (Caravaca and Rodán 2003; Semrany et al. 2012).

This accounts for the differences between the number of microorganisms and their diversity in loamy sand and in sandy loam. In addition, the varied response of autochthonous soil microorganisms to the tested diesel fuel might have resulted from the succession of microorganisms (Vazquez et al. 2013; Wu et al. 2014). New populations of bacteria and fungi may appear, which, due to intense metabolism, are capable of degrading PAHs and thus strongly affecting the soil microbiota (Semrany et al. 2012; Wyszkowska et al. 2015). According to Yemashova et al. (2007), the count of bacteria able to use petroleum carbohydrates as a source of energy in unpolluted soil is 10^2^–10^3^ in 1 g of soil, while in polluted soil, it equals 10^6^–5 × 10^7^ CFU g^−1^. The dominant bacteria which effectively participate in decomposition of crude oil are as follows: *Acinetobacter*, *Bacillus*, *Pseudomonas* and *Staphylococcus* (Fatima et al. 2015; Moscoso et al. 2012; Mnif et al. 2015), and the fungi with such an ability include *Aspergillus* and *Candida* (El-Hanafy et al. 2017; Fan et al. 2014; Silva et al. 2015). In general, changes in the communities of microorganisms are periodic, which is evidenced by fluctuations in the structure of r-strategy and K-strategy microorganisms (De Leij et al. 1993; Dorodnikov et al. 2009; Ernebjerga and Kishony 2012). In soils contaminated with petroleum products, fast-growing (r-strategy) microorganisms begin to dominate. After successful soil remediation, it can be expected that the state of equilibrium between these assemblages will be restored (De Leij et al. 1993; Dorodnikov et al. 2009; Ernebjerga and Kishony 2012). A stimulating effect of diesel oil on the number of copiotrophic, copiotrophic spore-forming and oligotrophic microorganisms, as well as actinobacteria, and a negative effect on cellulolytic bacteria and *Azotobacter* were observed by Wyszkowska and Kucharski (2005). Changes in the species composition of microorganism in the soil environment in terms of the fertility of soil are an adverse phenomenon. Changes to the number of microorganisms, which were observed in the authors’ own study in soil contaminated with Ekodiesel Ultra fuel, did not translate, however, into the resistance of microorganisms to the effects of DO, since both the stimulation and the inhibition of the proliferation of microorganisms by DO indicate the absence of their resistance. Even though the identification of the relationships between the indices describing the resistance (RS) of microorganisms is complicated and determined by numerous factors (Orwin and Wardle 2004), the determination in a study of the index of resistance enabled drawing objective conclusions on the stability of soil subjected to the effect of DO. The relatively low resistance of the tested microorganisms persisted for the entire duration of the study. It was lower with greater contamination with DO, yet definitely higher in loamy sand than in sandy loam. This is logical since differences in the abundance and bioavailability of elements are determined by, inter alia, the contents of clay fraction and the silt fraction in soil (Wyszkowska and Kucharski 2005). In the authors’ own study, the contents of silt and clay in sandy loam were more than two times greater than in loamy sand (Table 2). The change in oxygenation of soils resulting from the effect of DO is also of significance. The latter factor probably determined the clearly negative effects of DO on bacteria of the *Azotobacter* genus and on fungi. For *Azotobacter*, many aromatic hydrocarbons are a source of carbon and energy, while for PAHs contained in DO, such an effect was not observed. According to Semrany et al. (2012), partial or total inhibition of aerobic microorganisms’ metabolism results from the poor solubility of these substances in water.

Oil products are contaminants hazardous to the soil environment, which cause long-term changes to the reactions taking place in soil. They also deteriorate their physical and chemical properties (Caravaca and Rodán 2003; Semrany et al. 2012). While penetrating into the soil profile, they cause changes to the oxide reduction potential of soils (Griffiths and Philippot 2013) and soil lumping may occur. In the authors’ own study, the contamination of loamy sand and sandy loam with diesel fuel modified the physicochemical properties to a small extent. The exception was the significant increase in the soil content of organic carbon. Favourable effects of DO on the increase in organic carbon content were reported by Wyszkowska and Kucharski (2005) and for fuel oil by Kucharski and Jastrzębska (2005). An increase in organic carbon, total nitrogen and available potassium contents as well as an increase in the sorption capacity in contaminated soil was demonstrated by Wyszkowska et al. (2015). The relatively modest effect of diesel fuel on the remaining physicochemical properties in the presented study is probably due to the fact that the deposition of the oil product was one-off and occurred at the time of the establishment of the experiment.

In conclusion, it should be stressed, based on the conducted studies and the literature (Lipińska et al. 2015; Wu et al. 2014; Wyszkowska et al. 2015), that oil products significantly affect the stability of the soil ecosystem. It should also be noted that the long-term exposure of a particular ecosystem to stimuli of different types leads to the development of appropriate defensive mechanisms that are capable of maintaining the proper soil biological balance (Griffiths and Philippot 2013), as even the most stable substances in the soil environment can be metabolised by microorganisms (Adam et al. 2017; Fan et al. 2014; Fatima et al. 2015; El-Hanafy et al. 2017). Even though oil-derived hydrocarbons may be degraded by bacteria, fungi, yeasts and algae, it is bacteria that perform an essential role in the transformation of PAHs (Table 1). Biodegradation of PAHs in the soil environment is more often carried out by a consortium of microorganisms than by a particular species (Jung et al. 2016; Yergeau et al. 2012). Therefore, it is very important to identify the response of autochthonous microorganisms occurring in natural soil ecosystems on particular contaminants.

### Effect of diesel oil and soil physicochemical properties on soil enzymes

The stability of soil ecosystems is very well described by enzymatic activity, which is a sensitive indicator of changes in soil quality taking place in real time (Burns et al. 2013). The most important enzymes are those involved in the element cycle and in the degradation of chemical substances. The most reliable indicators used for estimating the quality of soils are considered to be dehydrogenases (the opinion of 28% of researchers), phosphatase (28%), β-glucosidase (16%) and urease (11%) (Gil-Sotres et al. 2005). Also in our own study, Ekodiesel Ultra fuel, while disturbing the metabolic profile of soil, changed the activity of enzymes involved in the processes of transformation of carbon, nitrogen, phosphorus and sulphur. The change in biochemical properties caused by the tested contaminant was reflected in the impact factors of diesel fuel on particular enzymes. The identification of these indicators enabled an objective conclusion as to whether the tested ecosystem is stable and capable of maintaining proper homeostasis. The factors of DO impact on the activity of all soil enzymes with the exception of catalase had negative values. These indices highlighted the disturbances to the metabolic profile of soil. On average, irrespective of the granulometric composition of soil or the duration of the effect of Ekodiesel Ultra oil on the reactions taking place in the soil, the enzymes may be ranked, in terms of their sensitivity to Ekodiesel Ultra, as follows (from the most to the least sensitive): urease > dehydrogenases > arylsulphatase > alkaline sulphatase > β-glucosidase > acid phosphatase. A relatively high resistance of enzymes to diesel fuel was also obtained by Kucharski and Jastrzębska (2005) and by Wyszkowska and Kucharski (2005). The changes to the biochemical activity of soils observed in this study were a response to biotic stress caused by contamination of soil with Ekodiesel ULTRA fuel. Certainly, the scale of changes to the biochemical activity of soils is determined by the type of oil product (Kucharski and Jastrzębska 2006; Wu et al. 2014; Wyszkowska et al. 2006). This is logical since these substances are a mixture of organic compounds with low bioavailability. The adverse effect of diesel fuel on the biochemical activity of soil is not a rare phenomenon. Serrano et al. (2009) also demonstrated the negative correlation between the contamination of soil with diesel fuel and the activity of dehydrogenase, arylsulphatase, protease, phosphatases and urease. On the other hand, β-glucosidase exhibited no significant response to the tested product. Wyszkowska et al. (2015) also found that β-glucosidase was the least resistant enzyme of the seven tested soil enzymes. In turn, according to Galiulin et al. (2012), the decomposition of oil-derived hydrocarbons increases in parallel with the increase in the activity of dehydrogenases which are directly involved in this process. The stimulating effect of diesel fuel and fuel oil on the activity of soil enzymes is indicated by the results of studies by Kucharski and Jastrzębska (2006) and by Wyszkowska et al. (2002a). Studies conducted by Wyszkowska et al. (2006) in soil contaminated with diesel fuel in doses ranging from 3 to 24 cm^3^ kg^−1^ of soil DM indicated an increase in the activity of dehydrogenases, urease and alkaline phosphatase. Wang et al. (2010) found that a mixture of hydrocarbons is particularly hazardous to the health of soil and such a mixture is present in oil products. According to the above-mentioned authors, hydrocarbons may both stimulate and inhibit the activity of phosphatases and catalase, although they are completely destructive to dehydrogenases, invertase and urease. In this study, the authors, in order to assess the quality of soil contaminated with Ekodiesel Ultra fuel as well as the activity of enzymes of the oxidoreductase and hydrolase classes, applied the index of biochemical activity (BA), which combines the activities of particular enzymes and more accurately reflects their responses to the tested contaminant. This index clearly demonstrated that disturbances to the soil metabolic profile were significant. Changes to the biochemical activity of soil result from the degree of degradation of Ekodiesel Ultra fuel, which was not only determined by the duration of DO retention in soil but also by the physicochemical properties of soil. In our study, it was loamy sand with pH_KCl_ = 6.70 and carbon content of 11.05 g C kg^−1^ of soil DM and sandy loam with pH_KCl_ = 6.80 and carbon content of 10.25 g C kg^−1^ of soil DM. Therefore, these were soils providing good conditions for the development of soil microbiota. This is important because certain microorganisms could use Ekodiesel Ultra fuel as a source of nutrients which, as a consequence, could affect the biosynthesis of enzymes by inducing or repressing phenomena. In this study, the activity of two enzymes, namely alkaline phosphatase (*r* = − 0.67, *P* = 0.017) and arylsulphatase (*r* = − 0.75, *P* = 0.005), was significantly negatively correlated with the N:P ratio. The same two enzymes were significantly negatively correlated with the C:P value (the activity of alkaline phosphatase at *r* = − 0.64, *P* = 0.025, and the activity of arylsulphatase at *r* = − 0.74, *P* = 0.006). The N:P ratio was positively correlated only with the activity of dehydrogenases (*r* = − 0.66, *P* = 0.020) and catalase (*r* = 0.81, *P* = 0.001). The activity of catalase was positively correlated with the CEC value (*r* = 0.66, *P* = 0.019), the same as the activity of alkaline phosphatase (*r* = 0.71, *P* = 0.009) and arylsulphatase (*r* = 0.63; *P* = 0.029). A significant positive correlation occurred between pH and the activity of alkaline phosphatase (*r* = − 0.71, *P* = 0.010) and arylsulphatase (*r* = − 0.74, *P* = 0.006), which resulted in negative coefficients of correlation between HAC and the activity of alkaline phosphatase (*r* = − 0.78, *P* = 0.003) and arylsulphatase (*r* = − 0.73, *P* = 0.007). To recapitulate, there was an unequivocal interaction between the activity of enzymes and the physicochemical properties of soil, which arose from the unstable conditions created in soil polluted with Ekodiesel Ultra fuel (Griffiths and Philippot 2013; Semrany et al. 2012; Wyszkowska and Kucharski 2005).

An increase in the soil content of PAHs is one of the factors contributing to the impact of diesel oil on soil enzymes. This study had demonstrated only a significant positive correlation between the activity of catalase and the PAH content, as well as a negative correlation between these hydrocarbons and the activity of dehydrogenases, urease and arylsulphatase. The contents of all tested PAHs were significantly higher in both tested soils contaminated with Ekodiesel Ultra fuel than in the non-contaminated soil. A high percentage of 2- and 3-ring PAHs in the total PAH content was noted. Similar relationships were also demonstrated in studies by Nganje et al. (2007) and by Wyszkowska et al. (2015). According to Baran and Oleszczuk (2001), phenanthrene and fluoranthene, i.e. 2–4-ring PAHs, account for 70% of the total contamination with PAHs. According to Wyszkowski and Ziółkowska (2013), the different PAH contents of soil contaminated with oil products depend on the type of contaminant, physicochemical properties of soil (principally organic substance content) and crop species. The effects of Ekodiesel Ultra fuel on PAH content were stronger in soil on which spring barley, yellow lupine and maize were cultivated, while the effects of petrol on PAH content were stronger in soil on which spring rape and oats were cultivated. Despite the significant increase in the aromatic hydrocarbon content of soils contaminated with Ekodiesel Ultra fuel and petrol, the contents of particular PAHs usually did not exceed 100 μg kg^−1^ of soil DM. Similar results were obtained in the authors’ own study.

## Conclusions

Disturbances in the reactions taking place in soil should be considered in terms of the possibility for restoring balance within the ecosystem. Therefore, the conducted experiment is an important link in the cycle of studies into the quality of the environment in which we live. The results presented in this study clearly indicate that the determination of the effects of oil products on the biochemical activity of soil is not a simple correlation. Significant differences were noted in the resistance of particular groups of microorganisms to the contamination of Endocalcaric Cambisol with Ekodiesel Ultra fuel. In loamy sand and sandy loam, the most resistant microorganisms were oligotrophic spore-forming bacteria. The resistance of microorganisms to contamination with Ekodiesel Ultra fuel was higher in loamy sand than in sandy loam. It decreased with the duration of exposure of microorganisms to the effects of Ekodiesel Ultra fuel. The effects of Ekodiesel Ultra fuel on the activity of particular enzymes varied. For dehydrogenases, urease, arylsulphatase and β-glucosidase, the impact factor IF_DO_ had negative values; for catalase, it had positive values, while for acid phosphatase and alkaline phosphatase, it was close to 0. A good measure of the effects of Ekodiesel Ultra fuel on the biochemical activity of soil was the BA index. In addition, there was a correlation between the degree of soil contamination and its PAH content. Additional data for the assessment of the condition of soils polluted with Ekodiesel Ultra fuel originated from the determination of interactions between the physicochemical properties of these soils and the soil microbiological activity. Therefore, the combination of microbiological and biochemical indicators with the parallel determination of physicochemical properties provides an opportunity to carry out a comprehensive analysis of soil quality under the pressure of oil-derived hydrocarbons.

## References

[CR1] Adam IKU, Duarte M, Pathmanathan J, Miltner A, Brüls T, Kästner M (2017). Microbial communities in pyrene amended soil–compost mixture and fertilized soil. AMB Expr.

[CR2] Agnello AC, Bagard M, van Hullebusch ED, Esposito G, Huguenot D (2016). Comparative bioremediation of heavy metals and petroleum hydrocarbons co-contaminated soil by natural attenuation, phytoremediation, bioaugmentation and bioaugmentation-assisted phytoremediation. Sci Total Environ.

[CR3] Akbari A, Ghoshal S (2015). Effects of diurnal temperature variation on microbial community and petroleum hydrocarbon biodegradation in contaminated soils from a sub-Arctic site. Environ Microbiol.

[CR4] Alef K, Nannipieri P, Alef K, Nannipieri P (1998). Methods in applied soil microbiology and biochemistry.

[CR5] Ameen F, Moslem M, Hadi S, Al-Sabri AE (2016). Biodegradation of diesel fuel hydrocarbons by mangrove fungi from Red Sea Coast of Saudi Arabia. Saudi J Biol Sci.

[CR6] Baran S, Bieliňska JE, Oleszczuk P (2004). Enzymatic activity in an airfield soil polluted with polycyclic aromatic hydrocarbons. Geoderma.

[CR7] Baran S, Oleszczuk P (2001). PAH content in Lublin soils due to the urban roadway system. Acta Agrophysica.

[CR8] Bento FM, Camargo FAO, Okeke BC, Frankenberger WT (2005). Diversity of biosurfactant producing microorganisms isolated from soils contaminated with diesel oil. Microbiol Res.

[CR9] Burns RG, DeForest JL, Marxsen J, Sinsabaugh RL, Stromberger ME, Wallenstein MD, Weintraub MN, Zoppini A (2013). Soil enzymes in a changing environment: current knowledge and future directions. Soil Biol Biochem.

[CR10] Canbolat MY, Bilen S, Çakmakçı R, Şahin F, Aydın A (2006). Effect of plant growth-promoting bacteria and soil compaction on barley seedling growth, nutrient uptake, soil properties and rhizosphere microflora. Biol Fertil Soils.

[CR11] Caravaca F, Rodán A (2003). Assessing changes in physical and biological properties in a soil contaminated by oil sludges under semiarid Mediterranean conditions. Geoderma.

[CR12] Chandran P, Das N (2011). Degradation of diesel oil by immobilized *Candida tropicalis* and biofilm form edongravels. Biodegradation.

[CR13] Chang KL, Ibrahim D, Ibrahim CO (2011). A laboratory scale bioremediation of tapis crude oil contaminated soil by bioaugmentation of *Acinetobacter baumannii* T30C. Afr J Microbiol Res.

[CR14] Chaudhary P, Sharma R, Singh SB, Nain L (2011). Bioremediation of PAH by *Streptomyces* sp. Bull Environ Contam Toxicol.

[CR15] COM (2006) Commission staff working document—accompanying document to the Communication from the Commission to the Council, the European Parliament, the European Economic and Social Committee and the Committee of the Regions—Thematic Strategy for Soil Protection—summary of the impact assessment

[CR16] Comber MHI, de Ferrer JA, Djemel N, Eadsforth CV, Lescrauwaet A, Léon Paumen M, Linington S, Redman A, Villalobos SA (2016) Analysis of N-, O-, and S-heterocyclics in petroleum products using GCxGC with specific detection. Concawe 75

[CR17] De Leij FAAM, Whipps JM, Lynch JM (1993). The use of colony development for the characterization of bacterial communities in soil and on roots. Microbiol Ecol.

[CR18] Díaz-Ramírez I, Escalante-Espinosa E, Schroeder RA, Fócil-Monterrubio R, Ramírez-Saad H (2013) Hydrocarbon biodegradation potential of native and exogenous microbial inocula in mexican tropical soils (eds. Rosenkranz F) Biodegradation of hazardous and special products: 155–178

[CR19] Dorodnikov M, Blagodatskaya E, BlagodatskyS FA, Kuzyakov Y (2009). Stimulation of r- vs. K-selected microorganisms by elevated atmospheric CO_2_ depends on soil aggregate size. FEMS Microbiol Ecol.

[CR20] Egner H, Riehm H, Domingo WR (1960). Untersuchun-gen über die chemische Bodenanalyse als Grundlage für die Beurteilung des Nährstoffzustandes der Böden. II. Chemische Extractionsmethoden zur Phospor- und Kaliumbestimmung. Ann Royal Agricult College Sweden.

[CR21] El-Hanafy AA, Anwar Y, Sabir JSM, Mohamed SA, Al-Garni SMS, Zinadah OSH, Ahmed MM (2017). Characterization of native fungi responsible for degrading crude oil from the coastal area of Yanbu, Saudi Arabia. Biotechnol Biotechnol Equip.

[CR22] Ernebjerga M, Kishony R (2012). Distinct growth strategies of soil bacteria as revealed by large-scale colony tracking. Appl Environ Microbiol.

[CR23] Fan MY, Xie RJ, Qin G (2014). Bioremediation of petroleum-contaminated soil by a combined system of biostimulation-bioaugmentation with yeast. Environ Technol.

[CR24] Fatima K, Afzal M, Imran A, Khan QM (2015). Bacterial rhizosphere and endosphere populations associated with grasses and trees to be used for phytoremediation of crude oil contaminated soil. Bull Environ Contam Toxicol.

[CR25] Fenglerowa W (1965). Simple method for counting *Azotobacter* in soil samples. Acta Microbiol Polon.

[CR26] Gałązka A, Król M, Perzyński A (2012). The efficiency of rhizosphere bioremediation with Azospirillum sp. and Pseudomonas stutzeri in soils freshly contaminated with PAHs and diesel fuel. Pol J Environ Stud.

[CR27] Galiulin RV, Bashkin VN, Galiulina RA (2012). Degradation of petroleum hydrocarbons in soil under the action of peat compost. Solid Fuel Chem.

[CR28] Galiulin RV, Galiulina RA (2015). Remediation of polar ecosystems polluted by gas condensate and oil hydrocarbons by biological preparations. Open Ecol J.

[CR29] Gargouri B, Mhiri N, Karray F, Aloui F, Sayadi S (2015). Isolation and characterization of hydrocarbon-degrading yeast strains from petroleum contaminated industrial wastewater. BioMed Res Int ID.

[CR30] Gil-Sotres FC, Trasar-Cepeda MC, Leiro’s S (2005). Different approaches to evaluating soil quality using biochemical properties. Soil Biol Biochem.

[CR31] Global Environment Outlook GEO_5_ (2012) Environment for the future we want. United Nations Environment Programme: 551

[CR32] Griffiths BS, Philippot L (2013). Insights into the resistance and resilience of the soil microbial community. FEMS Microbiol Rev.

[CR33] ISO 10390 (2005) Soil quality—determination of pH

[CR34] ISO 11261 (1995). Soil quality—determination of total nitrogen—modified Kjeldahl method.

[CR35] ISO 18287:2006. Soil quality—determination of polycyclic aromatic hydrocarbons (PAH)—gas chromatographic method with mass spectrometric detection (GC-MS)

[CR36] IUSS Working Group WRB: World Reference Base for Soil Resources (2014). International soil classification system for naming soils and creating legends for soil maps. WRB.

[CR37] Jung J, Philippot L, Park W (2016). Metagenomic and functional analyses of the consequences of reduction of bacterial diversity on soil functions and bioremediation in diesel-contaminated microcosms. Sci Rep.

[CR38] Kaczyńska G, Borowik A, Wyszkowska J (2015). Soil dehydrogenases as an indicator of contamination of the environment with petroleum products. Water Air Soil Pollut.

[CR39] Klute A (1996) Methods of soil analysis. Madison: American Society of Agronomy Agronomy Monograph 9

[CR40] Knight TR, Dick RP (2004). Differentiating microbial and stabilized β-glucosidase activity relative to soil quality. Soil Biol Biochem.

[CR41] Kucharski J, Jastrzębska E (2005). Effects of heating oil on the count of microorganisms and physico-chemical properties of soil. Pol J Environ St.

[CR42] Kucharski J, Jastrzębska E (2006). Effect of heating oil on the activity of soil enzymes and the yield of yellow lupine. Plant Soil Environ.

[CR43] Lipińska A, Kucharski J, Wyszkowska J (2013). Urease activity in soil contaminated with polycyclic aromatic hydrocarbons. Pol J Environ St.

[CR44] Lipińska A, Kucharski J, Wyszkowska J (2014). The effect of polycyclic aromatic hydrocarbons on the structure of organotrophic bacteria and dehydrogenase activity in soil. Polycycl Aromat Comp.

[CR45] Lipińska A, Kucharski J, Wyszkowska J (2014). Activity of arylsulphatase in soil contaminated with polycyclic aromatic hydrocarbons. Water Air Soil Pollut.

[CR46] Lipińska A, Wyszkowska J, Kucharski J (2015). Microbiological diversity and biochemical activity of soil contaminated with PAHs. Environ Sci Pollut Res.

[CR47] Martin J (1950). Use of acid rose bengal and streptpmycin in the plate method for estimating soil fungi. Soil Sci.

[CR48] Mnif I, Mnif S, Sahnoun R, Maktouf S, Ayedi Y, Ellouze-Chaabouni S, Ghribi D (2015). Biodegradation of diesel oil by a novel microbial consortium: comparison between co-inoculation with biosurfactant-producing strain and exogenously added biosurfactants. Environ Sci Pollut Res.

[CR49] Moeskops B, Buchan D, Sleutel S, Herawaty L, Husen E, Saraswati R, Setyorini D, De Neve S (2010). Soil microbial communities and activities under intensive organicand conventional vegetable farming in West Java, Indonesia. Appl Soil Ecol.

[CR50] Moscoso F, Teijiz I, Deive FJ, Sanromán MA (2012). Efficient PAHs biodegradation by a bacterial consortium at flask and bioreactor scale. Bioresour Technol.

[CR51] Mulder EG, Antheumisse J (1963). Morphologie, physiologie et ecologie des *Arthrobacter*. Ann Inst Pasteur.

[CR52] Nelson DW, Sommers LE (1996) Total carbon, organic carbon, and organic matter. In: D.L. Sparks (Ed.) Method of soil analysis: chemical methods, American Society of Agronomy (pp. 1201–1229), Madison, WI

[CR53] Nganje TN, Edet AE, Ekwere SJ (2007). Distribution of PAHs in surface soils from petroleum handling facilities in Calabar. Environ Monit Assess.

[CR54] Niepceron M, Martin-Laurent F, Crampon M, Portet-Koltalo F, Akpa-Vinceslas M, Legras M, Bru D, Bureau F, Bodilis J (2013). GammaProteobacteria as a potential bioindicator of a multiple contamination by polycyclic aromatic hydrocarbons (PAHs) in agricultural soils. Environ Pollut.

[CR55] Öhlinger R, Schinner F, Ohlinger R, Kandler E, Margesin R (1996). Dehydrogenase activity with the substrate TTC. Methods in soil biology.

[CR56] Ohta H, Hattori T (1983). Oligotrophic bacteria on organic debris and plant roots in paddy field. Soil Biol Biochem.

[CR57] Orwin KH, Wardle DA (2004). New indices for quantifying the resistance and resilience of soil biota to exogenous disturbances. Soil Biol Biochem.

[CR58] Park IS, Park JW (2011). Determination of a risk management primer at petroleum-contaminant sites: developing new human health risk assessment strategy. J Hazard Mater.

[CR59] Parkinson D, Gray FRG, Williams ST (1971) Methods for studying the ecology of soil micro-organism. Blackwell Scientific Publication, Oxford, IBP Handbook 19

[CR60] Pasumarthi R, Chandrasekaran S, Mutnuri S (2013). Biodegradation of crude oil by *Pseudomonas aeruginosa* and *Escherichia fergusonii* isolated from the Goan coast. Mar Pollut Bull.

[CR61] Patel JG, Kumar JIN, Kumar RN, Khan SR (2016). Biodegradation capability and enzymatic variation of potentially hazardous polycyclic aromatic hydrocarbons—anthracene and pyrene by anabaena fertilissima. Polycycl Aromat Comp.

[CR62] Perez-Leblic MI, Turmero A, Hernandez M, Hernandez AJ, Pastor J, Ball AS, Rodrigues J, Arias ME (2010). Influence of xenobiotic contaminants on landfill soil microbial activity and diversity. J Environ Manag.

[CR63] Pinedo J, Ibáñez R, Irabien A (2012). Risk assessment of total petroleum hydrocarbons (TPHs) fractions. Chem Engineer Transact.

[CR64] PN-R-04032 (1998) Soil and mineral materials—sampling and determination of particle size distribution

[CR65] Ramkumar S, Kirubakaran V (2016). Biodiesel from vegetable oil as alternate fuel for C.I engine and feasibility study of thermal cracking: a critical review. Energy Convers Manag.

[CR66] Ros M, Rodriguez I, Garcia C, Hernandez T (2010). Microbial communities involved in the bioremediation of an aged recalcitrant hydrocarbon polluted soil by using organic amendments. Bioresour Technol.

[CR67] Schlichting E, Blume HP, Stahr K (1995) Bodenkundliches Praktikum. Pareys Studientexte 81, Berlin: Blackwell Wissenschafts-Verlag

[CR68] Semrany S, Faviera L, Djelal H, Tahac S, Amranea A (2012). Bioaugmentation: possible solution in the treatment of bio-refractory organic compounds (Bio-ROCs). Biochem Eng J.

[CR69] Serrano A, Tejada M, Gallego M, Gonzalez JL (2009). Evaluation of soil biological activity after a diesel fuel spill. Sci Total Environ.

[CR70] Shrestha RA, Pham TD, Sillanpää M (2010). Electro ultrasonic remediation of polycyclic aromatic hydrocarbons from contaminated soil. J Appl Electrochem.

[CR71] Silva DP, Cavalcanti DD, de Melo EV, dos Santos PNR, da Luz ELP, de Gusmao NB, Sousa MF (2015). Bio-removal of diesel oil through a microbial consortium isolated from a polluted environment. Int Biodeterior Biodegr.

[CR72] Sivitskaya V, Wyszkowski M (2013). Changes in the content of some macroelements in maize (Zea mays L.) under effect of fuel oil after application of different substances to soil. J Elem.

[CR73] Soleimani M, Afyuni M, Hajabbasi MA, Nourbakhsh F, Sabzalian MR, Christensen JH (2010). Phytoremediation of an aged petroleum contaminated soil using endophyte infected and non-infected grasses. Chemosphere.

[CR74] Soltani N, Baftechi L, Dezfulian M, Shokravi S, Alnajar N (2012). Molecular and morphological characterization of oil polluted microalgae. Int J Environ Res.

[CR75] Sood N, Lal B (2009). Isolation of a novel yeast strain Candida digboiensis TERI ASN6 capable of degrading petroleum hydrocarbons in acidic conditions. J Environ Manag.

[CR76] Statsoft, Inc., Statistica (2015) (data analysis software system), version 12.5. www.statsoft.com

[CR77] Subhani A, Changyong H, Zhengmiao Y, Min L, El-Ghamry A (2001). Impact of soil environment and agronomic practices on microbial/dehydrogenase enzyme activity in soil. A review. Pak J Biol Sci.

[CR78] Thavamani P, Megharaj M, Naidu R (2012). Bioremediation of high molecular weight polyaromatic hydrocarbons co-contaminated with metals in liquid and soil slurries by metal tolerant PAHs degrading bacterial consortium. Biodegradation.

[CR79] Vazquez S, Nogales B, Ruberto L, Mestre C, Christiee-Oleza J, Ferrero M, Bosch R, Mac Cormack WP (2013). Characterization of bacterial consortia from diesel-contaminated Antarctic soils: towards the design of tailored formulas for bioaugmentation. Int Biodeterior Biodegr.

[CR80] Vong PC, Piutti S, Slezack-Deschaumes S, Beniziri E, Guckert A (2008). Sulphur immobilization and arylsulphatase activity in two calcareous arable and fallow soils as affected by glucose additions. Geoderma.

[CR81] Wald J, Hroudova M, Jansa J, Vrchotova B, Macek T, Uhlik O (2015). Pseudomonads rule degradation of polyaromatic hydrocarbons in aerated sediment. Front Microbiol.

[CR82] Wang J, Zhan X, Zhou L, Lin Y (2010). Biological indicators capable of assessing thermal treatment efficiency of hydrocarbon mixture-contaminated soil. Chemosphere.

[CR83] Wang X, Cai Z, Zhou Q, Zhang Z, Chen C (2012). Bioelectrochemical stimulation of petroleum hydrocarbon degradation in saline soil using U-tube microbial fuel cells. Biotechnol Bioeng.

[CR84] Winogradski S (1953). Soi lmicrobiology (in Polish).

[CR85] Wu B, Lan T, Lu D, Liu Z (2014). Ecological and enzymatic responses to petroleum contamination. Environ Sci Process Impacts.

[CR86] Wyszkowska J, Borowik A, Kucharski J (2015). Response of Avena sativa, microorganisms and enzymes to contamination of soil with diesel oil. Plant Soil Environ.

[CR87] Wyszkowska J, Borowik A, Kucharski M, Kucharski J (2013). Applicability of biochemical indices to quality assessment of soil polluted with heavy metals. J Elem.

[CR88] Wyszkowska J, Kucharski J (2005). Correlation between the number of cultivatable microorganisms and soil contamination with diesel oil. Pol J Environ St.

[CR89] Wyszkowska J, Kucharski J, Wałdowska E (2002). The influence of diesel oil contamination on soil enzymes activity. Rost Vyr.

[CR90] Wyszkowska J, Kucharski J, Wałdowska E (2002). The influence of diesel oil contamination on soil microorganisms and oat growth. Rost Vyr.

[CR91] Wyszkowska J, Kucharski M, Kucharski J (2006). Application of the activity of soil enzymes in the evaluation of soil contamination by diesel oil. Pol J Environ St.

[CR92] Wyszkowska J, Wyszkowski M (2010). Activity of soil dehydrogenases, urease and acid and alkaline phosphatase in soil polluted with petroleum. J Toxic Environ Health Part A.

[CR93] Wyszkowski M, Ziółkowska A (2013). Content of polycyclic aromatic hydrocarbons in soils polluted with petrol and diesel oil after remediation with plants and various substances. Plant Soil Environ.

[CR94] Yemashova NA, Murygina VP, Zhukov DV, Zakharyantz AA, Gladchenko MA, Appanna V, Kalyuzhnyi SV (2007). Biodeterioration of crude oil and oil derived products: a review. Rev Environ Sci Biotechnol.

[CR95] Yengejeh JR, Sekhavatjou MS, Maktabi P, Arbab Soleimani N, Khadivi S, Pourjafarian V (2014). The biodegradation of crude oil by Bacillus subtilis isolated from contaminated soil in hot weather areas. Int J Environ Res.

[CR96] Yergeau E, Sanschagrin S, Beaumier D, Greer CW (2012). Metagenomic analysis of the bioremediation of diesel-contaminated Canadian high Arctic soils. PLoS One.

[CR97] Yuan Y, Guo S, Fengmei L, Wu B, Yang X, Li X (2016). Coupling electrokinetics with microbial biodegradation enhances the removal of cycloparaffinic hydrocarbons in soils. J Hazard Mater.

[CR98] Zhan X, Wu W, Zhou L, Liang J, Jiang T (2010). Interactive effect of dissolved organic matter and phenanthrene on soil enzymatic activities. J Environ Sci.

[CR99] Ziółkowska A, Wyszkowski M (2010). Toxicity of petroleum substances to microorganisms and plants. Ecol Chem Eng S.

